# Different Inspiratory Flow Waveform during Volume-Controlled Ventilation in ARDS Patients

**DOI:** 10.3390/jcm10204756

**Published:** 2021-10-17

**Authors:** Davide Chiumello, Andrea Meli, Tommaso Pozzi, Manuela Lucenteforte, Paolo Simili, Elda Sterchele, Silvia Coppola

**Affiliations:** 1 Department of Anesthesia and Intensive Care, ASST Santi Paolo e Carlo, San Paolo University Hospital, Via Di Rudini 9, 20142 Milan, Italy; silvia_coppola@libero.it; 2 Department of Health Sciences, University of Milan, 20142 Milan, Italy; tommaso.pozzi94@gmail.com (T.P.); manuela.lucenteforte@unimi.it (M.L.); paolo.simili@unimi.it (P.S.); eldadiletta.sterchele@gmail.com (E.S.); 3 Coordinated Research Center on Respiratory Failure, University of Milan, 20142 Milan, Italy; 4 Department of Anesthesia, Intensive Care and Emergency, Fondazione IRCCS Ca’ Granda Hospital Maggiore Policlinico, Via F. Sforza 35, 20122 Milan, Italy; andreameli24@gmail.com

**Keywords:** acute respiratory distress syndrome, mechanical ventilation, flow waveforms

## Abstract

The most used types of mechanical ventilation are volume- and pressure-controlled ventilation, respectively characterized by a square and a decelerating flow waveform. Nowadays, the clinical utility of different inspiratory flow waveforms remains unclear. The aim of this study was to assess the effects of four different inspiratory flow waveforms in ARDS patients. Twenty-eight ARDS patients (PaO_2_/FiO_2_ 182 ± 40 and PEEP 11.3 ± 2.5 cmH_2_O) were ventilated in volume-controlled ventilation with four inspiratory flow waveforms: square (SQ), decelerating (DE), sinusoidal (SIN), and trunk descending (TDE). After 30 min in each condition, partitioned respiratory mechanics and gas exchange were collected. The inspiratory peak flow was higher in the DE waveform compared to the other three waveforms, and in SIN compared to the SQ and TDE waveforms, respectively. The mean inspiratory flow was higher in the DE and SIN waveforms compared with TDE and SQ. The inspiratory peak pressure was higher in the SIN and SQ compared to the TDE waveform. Partitioned elastance was similar in the four groups; mechanical power was lower in the TDE waveform, while PaCO_2_ in DE. No major effect on oxygenation was found. The explored flow waveforms did not provide relevant changes in oxygenation and respiratory mechanics.

## 1. Introduction

Invasive positive pressure mechanical ventilation is the mainstay of respiratory support during acute respiratory failure [1]. Mechanical ventilation (MV) is able to replace the pump function of the respiratory system, aiming to guarantee tidal ventilation and, possibly, adequate gas exchange. In ARDS, invasive mechanical ventilation is used in up to 33% of patients [2,3], where the two most common used types of MV are volume-controlled (VCV) and pressure-controlled ventilation (PCV). Indeed, VCV is used in approximately 60% of patients [2,3].

During VCV, the ventilator delivers a preset tidal volume with a typical square flow waveform, whereas during PCV, the ventilator produces an appropriate flow (i.e., a decelerating flow waveform) to reach the set inspiratory pressure [4]. Theoretically, the decelerating flow compared to the constant flow (square waveform) should optimize the lung inflation and oxygenation by allowing for a longer inspiratory time. This would result in a longer time for gas to access those alveolar units that are characterized by a prolonged time constant [5]. It has been found that pressure-controlled compared to volume-controlled ventilation significantly reduced peak airway pressure, improved lung compliance, and avoided a regional overdistension via a more homogeneous gas distribution [6]. However, a recent review was not able to find any difference between PCV and VCV in terms of clinical outcome during acute respiratory failure [7].

Thus, it has been suggested that the employment of a modified VCV, where the set volume is delivered through a decelerating flow waveform, could join the benefits of a fixed tidal volume with a theoretical improvement in gas exchange [7]. Indeed, both in ARDS and in post-operative patients, modified VCV with a decelerating flow was found to provide better oxygenation and an improvement in terms of lung compliance compared to the ‘classical’ VCV (square flow waveform) [8,9,10,11]. On the contrary, previous animal and human data reported conflicting results regarding the use of different inspiratory flow patterns during controlled mechanical ventilation [11,12,13,14,15,16].

However, in the cited studies, old generation mechanical ventilators were employed and, more importantly, the modifications of the inspiratory flow waveforms were also associated to changes in tidal volume, respiratory rate, and inspiratory time [4,9,10,12,13,16]. In addition, there is a paucity of data regarding the possible utility of the decelerating flow waveform in terms of reducing ventilator-induced lung injury (VILI). Recently, mechanical power has been suggested as a reliable indicator of VILI at the bedside [17]. Yet, at the present time, the clinical utility of the different inspiratory flow waveforms both in terms of gas exchange and in mechanical power remains unclear.

Therefore, we conducted a prospective randomized study to assess the effects of four different inspiratory flow waveforms in ARDS patients during controlled MV.

## 2. Materials and Methods

This prospective study was conducted from July 2019 to February 2021 in the general intensive care unit of the ASST Santi Paolo e Carlo, San Paolo Hospital, Milan. The protocol was approved by the local ethical committee (N 2018/ST/274) and informed consent was obtained according to Italian regulations. All consecutive patients with ARDS according to the Berlin definition criteria were enrolled [18]. Exclusion criteria included age less than 18 years, barotrauma, hemodynamic instability, and a history of chronic obstructive pulmonary disease (COPD).

### 2.1. Study Protocol

Sedation with Propofol and Remifentanyl and neuromuscular blocking agents were provided to obtain a Richmond Agitation-Sedation Scale (RASS) value of −5. Patients were all ventilated with the same mechanical ventilator (Hamilton S1 ventilator, Hamilton Medical Inc., Bonaduz, Switzerland) with a tidal volume (TV) of 7–9 mL/kg of the predicted body weight (PBW) with a constant flow. The Positive End-Expiratory Pressure (PEEP) was set following the high-PEEP arm of the LOVS study [19]. The ratio of inspiratory time to expiratory time (I:E ratio) was fixed by the operator for all patients 1:2.5, while respiratory rate was fixed within each patient to reach and maintain normocapnia.

**Figure 1:**
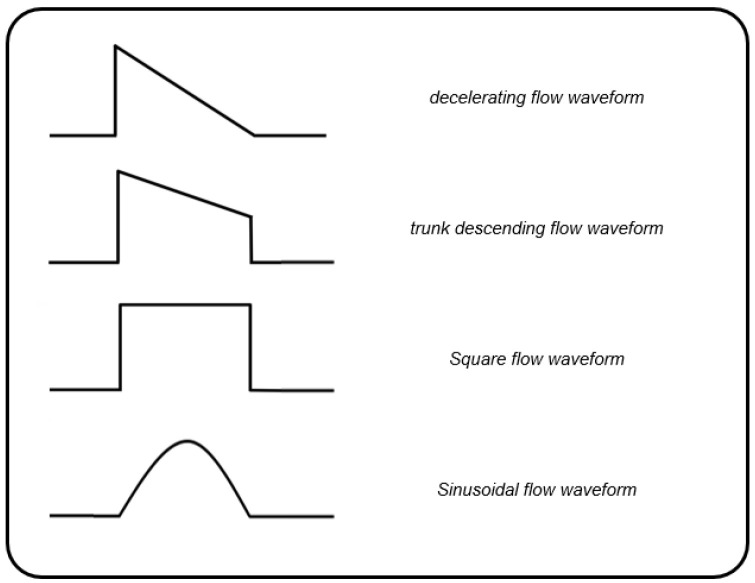
Schematic representation of the delivered inspiratory flow waveforms.

After 1 h of mechanical ventilation with a set tidal volume, respiratory rate, and PEEP, the patients received in a randomized fashion four inspiratory flow waveforms: square (SQ), 100% decelerating (DE), sinusoidal (SIN), and 50% trunk descending (TDE) (Figure 1).

**Figure 2:**
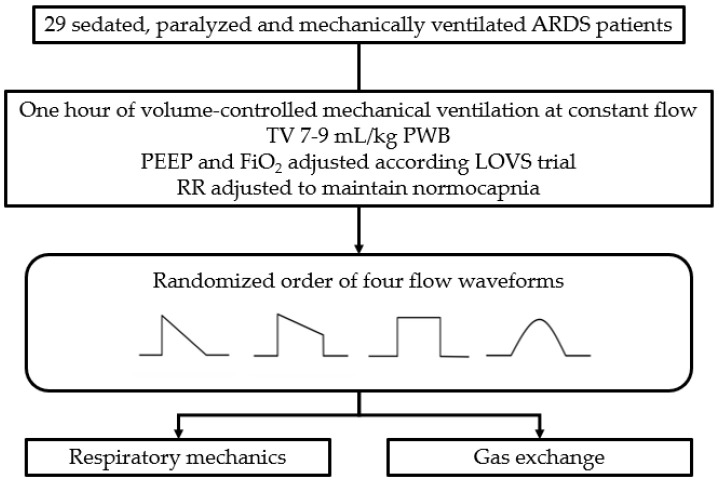
Study flow-chart.

After 30 min in each step, respiratory mechanics and gas exchange data were collected and subsequently analyzed (Figure 2).

### 2.2. Measurements

The inspiratory flow rate was measured with a heated pneumotachograph (Fleisch no. 2, Fleisch, Lausanne, Switzerland). Airway pressure was measured proximally to the endotracheal tube with a dedicated pressure transducer (MPX 2010 DP. Motorola, Solna, Sweden). Esophageal pressure was measured using a standard balloon catheter (Smart Cath, Viasys, Palm Springs, CA, USA) consisting of a 103 cm tube with an external diameter of 3 mm and a thin-walled balloon 10 cm long. The esophageal catheter was emptied of air and introduced trans-orally into the esophagus to reach the stomach at a depth of 50–55 cm from the mouth. Subsequently, the balloon was inflated with 1.5 mL of air. The intragastric position of the catheter was confirmed by a positive pressure deflection of intra-abdominal pressure during an external manual epigastric pressure. At last, the catheter was retracted and positioned in the low esophageal position. The amount of gas in the balloon was periodically checked throughout the experiment.

All traces were sampled at 100 Hz and processed on a dedicated data acquisition system (Colligo and Computo, Via Francesco Sforza 35, Milan, Italy, www.elekton.it (accessed on 5 August 2021)) [20].

In the last two minutes of each measurement period, consecutive breaths were recorded to collect the respiratory rate, tidal volume, inspiratory peak flow, and mean inspiratory flow.

Total breathing time was calculated from the ratio between 60 s and the respiratory rate.

In the last 1 min of each study period, a series of two end-inspiratory and end-expiratory pauses were performed. Respiratory system, lung, and chest wall elastance were computed according to standard formulae [21].

The end expiratory lung gas volume (EELV) was measured by a simplified helium dilution technique during an end-expiratory pause at PEEP [20].

Mechanical power is the result of the energy delivered from the ventilator to the respiratory system multiplied by the respiratory rate, and is expressed in J/min; it was calculated according to Gattinoni et al. [17]:

Mechanical Power = 0.098 × Respiratory Rate × Tidal Volume × (Airway Peak Pressure −0.5 × (Airway Plateau Pressure–PEEP)).

### 2.3. Statistical Analysis

The Shapiro–Wilk test was used to test the normality of distribution of continuous variables. Data are reported as mean (standard deviation) or median [interquartile range].

The analysis of variance (ANOVA) for repeated measures was used to compare data of the four groups in case on normal distribution; otherwise, the Friedman test was employed. Student’s *t*-test or Wilcoxon–Mann–Whitney test were used for pairwise comparisons, as appropriate. Fisher’s exact test was used for categorical variables.

For sample size calculation, we expected a maximal variation of PaO_2_/FiO_2_ of 15% between the four groups, assuming baseline values described in severe ARDS as 75.0 ± 9.5. With a two-sided statistical significance of 0.05, a sample size of 28 subjects would be enough to elicit a power of 0.90; a post-hoc power analysis confirmed the adequacy of the study population with respect to the selected statistical power cutoff.

The statistical significance cut off was considered as a *p* value < 0.05. The statistical analysis was performed using RStudio (R Foundation for Statistical Computing, Vienna, Austria).

## 3. Results

Twenty-eight patients were enrolled, and the baseline characteristics are shown in Table 1. The patients were randomized after a median of 1 [1,2] days from intensive care admission. Twenty patients (71%) presented a pulmonary form of ARDS; the PaO_2_/FiO_2_ ratio and PEEP were 182 ± 40 and 11.3 ± 2.5 cmH_2_O, respectively. The mean applied tidal volume for predicted body weight was 8.7 ± 1.5 mL/Kg. The intensive care mortality was 43%.

### 3.1. Breathing Pattern and Respiratory Mechanics

**Figure 3:**
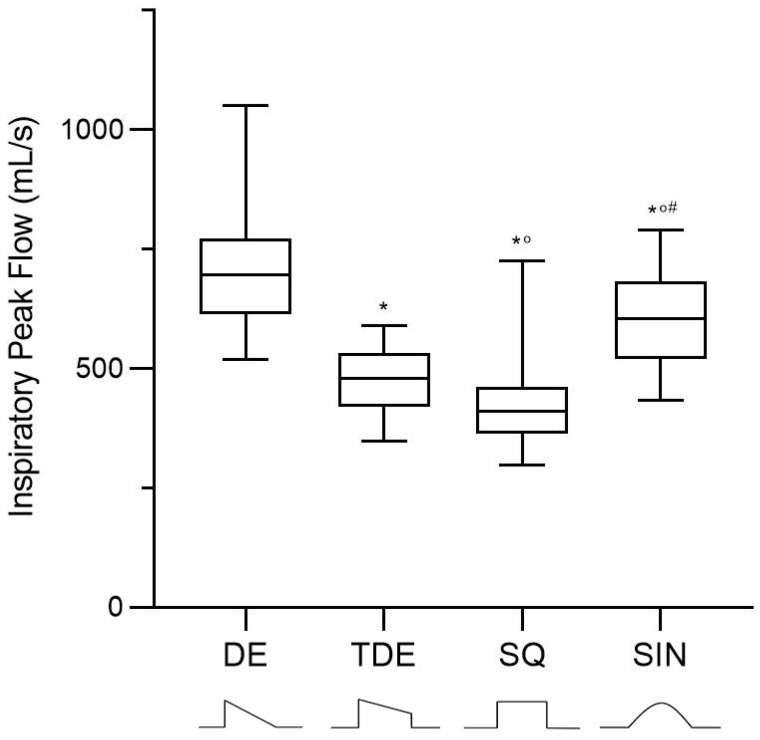
Inspiratory peak flow comparison among the four flow waveforms. DE: Decelerating Waveform; TDE: Trunk Decelerating Waveform; SQ: Square Waveform; SIN: Sinusoidal Waveform. *: vs. DE; °: vs. TDE; #: vs. SQ. *p* < 0.05.

The respiratory rate and tidal volume were unchanged throughout the four tested conditions (Table 2). The mean applied PEEP was 11.2 ± 2.1 cmH_2_O. The inspiratory peak flow was higher in the decelerating compared to the other three waveforms and in the sinusoidal waveform compared to the square and trunk decelerating waveforms (Figure 3). The mean inspiratory flow was higher in the decelerating and sinusoidal waveform, compared with both the trunk decelerating and square ones.

**Figure 4:**
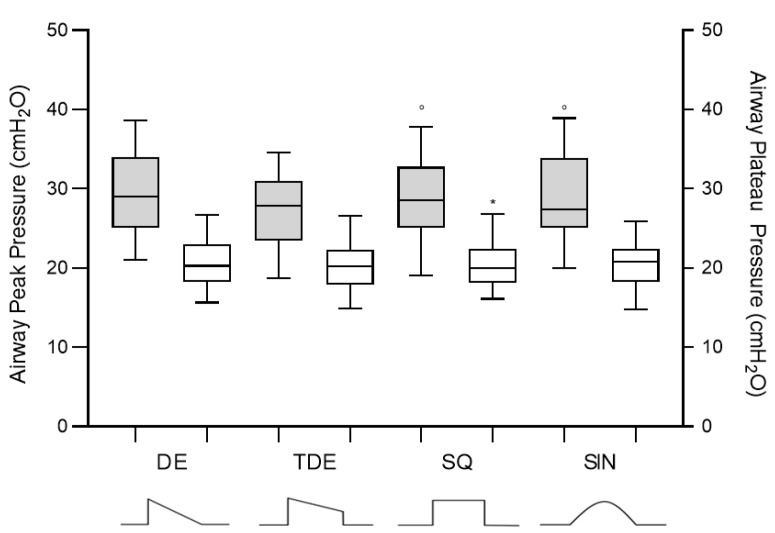
Comparison of inspiratory peak and plateau pressure among the four flow waveforms. DE: Decelerating Waveform; TDE: Trunk Decelerating Waveform; SQ: Square Waveform; SIN: Sinusoidal Waveform. *: vs. DE; °: vs. TDE; #: vs. SQ. *p* < 0.05.

The inspiratory time was not different among the tested flow waveforms, while inspiratory peak pressure was significantly higher in the sinusoidal and in the square compared to the trunk decelerated waveform (Table 3 and Figure 4).

The respiratory system elastance and lung elastance were similar among the flow waveforms. Similarly, end-expiratory lung gas volume remained unchanged. The mechanical power was slightly lower with the trunk decelerating waveform (Table 3).

### 3.2. Gas Exchange and Hemodynamics

No major effect on arterial oxygenation was observed according to the application of different waveforms. The square waveform resulted in a higher arterial carbon dioxide partial pressure compared to the decelerating one (Table 4).

## 4. Discussion

This study evaluated the application of different inspiratory flow waveforms during controlled mechanical ventilation in ARDS patients. Our results did not show major beneficial effects on the partitioned respiratory mechanics and arterial oxygenation between the four different groups. A slight reduction in arterial carbon dioxide and mechanical power was observed with the decelerating and the trunk decelerating waveform.

In the passive condition, during VCV, the ventilator delivers the preset tidal volume using the same flow waveform at every breath; thus, the resulting airway pressure depends on the ventilator circuit and on the mechanical characteristics of the respiratory system [6]. During VCV with a square waveform, the flow quickly rises linearly to the value set on the ventilator and then remains constant during inspiration until the tidal volume has been delivered; then, it falls rapidly before exhalation begins.

In the VCV setting, in addition to the possibility of changing the respiratory rate, inspiratory time, peak flow, and PEEP, at the present time, physicians are able to set the inspiratory flow pattern, especially when new generation mechanical ventilators are employed [1]. The most frequent waveforms available on the ventilators are the square, decelerating, and sinusoidal flow waveforms [1]. Over the years, several data have been published about the optimal flow waveform, though using old-generation mechanical ventilators [3,9,10,11,22,23]. The theoretical possible advantages of the different inspiratory flow patterns should be related to the difference in the ventilation distribution into the lung, resulting in a theoretical reduction in the inspiratory airway peak pressure, improvement of lung recruitment, and reduction of mechanical power and CO_2_ clearance [9,10,11,23].

In the present study, in sedated and paralyzed ARDS patients, we used a common mechanical ventilator to deliver VCV: four different flow waveforms were evaluated (square, sinusoidal, decelerating, and trunk decelerating).

When the square waveform is delivered, the peak inspiratory flow is equal to the mean inspiratory flow. On the other hand, during decelerating, trunk decelerating, and sinusoidal waveforms, the inspiratory flow is near zero at the end of inspiration.

According to the used ventilator, the inspiratory time remained unchanged in this study. Thus, the same volume of gas was delivered during the same inspiratory time of use while changing between the different flow waveforms. Thus, only the effects of different shapes and inspiratory flow during the same inspiratory time were evaluated.

When applying the decelerating waveform, which delivers the gas flow at a progressively slower rate, the peak inspiratory flow was higher compared to the square, trunk decelerating, and sinusoidal waveforms. However, this did not translate to major changes in the peak inspiratory and plateau pressure, suggesting that significant changes in the airway resistance or dynamic lung elastance did not take place. This was also reflected by the measurement of static lung elastance, which was similar among the different waveforms. Johansson et al., using a flow-controlled ventilator that uses linear inspiratory and expiratory flow, showed that the square compared to the decelerating waveform resulted in higher values of inspiratory peak pressure without any changes in the respiratory system compliance in a group of patients with acute respiratory failure [13]. Similarly, Guerin et al. found that the decelerating and square waveform did not change respiratory system compliance in sedated and paralyzed COPD patients where constant inspiratory time and tidal volume were maintained [12].

When the changes in flow waveform also affect the inspiratory time, the decelerating waveform is associated with a longer inspiratory time; this results in a significant decrement of the inspiratory peak pressure compared to the square and sinusoidal waveforms. Nevertheless, this is without any substantial change in respiratory compliance [10]. On the contrary, Al Saady et al. reported that the decelerating waveform significantly reduced the inspiratory peak pressure and plateau pressure compared to the square waveform [9]. A possible explanation for these conflictual results could be due to the high initial peak flow, which is then followed by a progressive decline with the decelerating waveforms, leaving more time for the gas distribution into the lung, which in turn can promote lung recruitment [9,10,11,22,23]. In addition, the presence of an end-inspiratory pause could further promote an additional lung recruitment effect [22]. Thus, discrepancies among the available studies are likely related to the different algorithms present on mechanical ventilators, which tend to modify the inspiratory time when the inspiratory flow waveform is changed.

Interestingly, high inspiratory flow could participate in VILI by exerting augmented stress on lung parenchyma, especially in those regions where short time constants do not allow for dissipation of the inspiratory forces when the insufflation is quick [24,25]. In this context, a higher peak inspiratory flow, such as those observed during decelerating or sinusoidal waveforms, might be more harmful than square waveforms, especially in an inhomogeneous parenchyma that can be found in ARDS patients. This hypothesis is supported by experimental literature, where a role of inspiratory flow has been recognized in the genesis of VILI [26,27,28,29,30].

In order to evaluate the possible VILI, we computed the mechanical power [17]. The mechanical power based on the motion equation, depends on the driving pressure, tidal volume, respiratory rate, peak pressure, and PEEP. The respiratory rate and tidal volume did not change throughout the study, thus the mechanical power resulted from the interaction of the peak pressure and driving pressure. The lowest mechanical power was obtained with the trunk decelerating waveform, while it was similar between the decelerating and square waveform.

During the early 1970s, several animal and human studies evaluated the effects of a variety of flow waveforms in gas exchange in an heterogenous population of mechanically ventilated patients [9,10,11,12,13,14,15,16]. This data showed inconsistent results mainly due to the small sample size, type of ventilator, and ventilatory settings. Kenneth et al. showed that the decelerating compared to the square waveform slightly improved the oxygenation from a mean of 75 ± 11 to 85 ± 11 mmHg, probably due to a higher mean airway pressure, which could have facilitated alveolar recruitment [11]. A similar increase of alveolar oxygenation was also found in 14 patients applying a decelerating compared to a square waveform [9].

All the cited studies were performed in well-sedated patients, with or without paralysis, thus no patient–ventilator asynchrony was involved. However, to improve patient–ventilator synchrony, among the different strategies, modification of the inspiratory flow has been suggested, thus these different flow wave forms could also play a role during difficult patient–ventilator interaction.

Our study is one of the few studies that has showed that by maintaining a constant tidal volume, PEEP, and inspiratory time, arterial oxygenation, intrapulmonary shunt, and dead space were equivalent among the different flow waveforms.

## 5. Limitations

A limitation of this study is that only one type of mechanical ventilator was used, thus it is not possible to generalize the present results to different ventilators. Yet, the use of the same ventilator for the whole study cuts the possibility of inter-patient variability due to technical aspects. Another limitation is the length of the stabilization period, which was restricted to 30 min. At last, the impact of the different inspiratory waveforms on mechanical power might be underestimated by the short follow-up period for each step, and thus it is worth studying in future investigations.

## 6. Conclusions

In this study, we observed that different flow waveforms during volume-controlled ventilation allow the ventilator to deliver significantly different peak inspiratory flows. Nevertheless, this did not translate to major changes in oxygenation or partitioned respiratory mechanics, except for the mechanical power and arterial carbon dioxide partial pressure, which was lower with the decelerating and trunk decelerating waveforms, respectively.

## Figures and Tables

**Table 1:** Characteristics of the study population.

Characteristics	Study Population (*n* = 28)
Age, years	72 (56–78)
Male sex, % (*n*)	71 (20)
Height, cm	168 (164–172)
Weight, kg	71 (66–89)
BMI, kg/m^2^	25 (22–31)
SAPS II	45 (35–51)
Origin of ARDS, % (*n*)	
Pulmonary Extrapulmonary	71 (20) 29 (8)
ARDS severity	
Mild, % (*n*) Moderate, % (*n*) Severe, % (*n*)	36 (10) 64 (18) 0 (0)
Duration of mechanical ventilation, days	15 (11–22)
Intensive care unit length of stay, days	16 (14–24)
Intensive care unit mortality, % (*n*)	
Dead Alive	43 (12) 57 (16)
Tidal volume, mL	458 (432–471)
Tidal volume per predicted body weight, mL/kg	8.7 (8.2–8.9)
Respiratory rate, bpm	16 (15–17)
Minute ventilation, L/min	7.2 (6.5–7.4)
PEEP, cmH_2_O	11.3 (10.4–11.5)
Driving pressure, cmH_2_O	10.6 (9.2–11.0)
Respiratory system Elastance, cmH_2_O/L	20.4 (16.0–21.6)
PaCO_2_, mmHg	51.5 (46.4–53.2)
PaO_2_, mmHg	72.8 (68.0–84.5)
FiO_2_, mmHg	0.4 (0.4–0.5)
PaO_2_/FiO_2_	182 (151–189)

Quantitative data are presented as mean ± SD or median (IQR), as appropriate; categorical variables are expressed as % (*n*). BMI: Body Mass Index; SAPS II: Simplified Acute Physiology Score II; PEEP: Positive End-Expiratory Pressure; PaCO_2_: carbon dioxide arterial partial pressure; PaO_2_: oxygen arterial partial pressure; PaO_2_/FiO_2_: oxygen arterial partial pressure on inspired fraction of oxygen ratio.

**Table 2:** Breathing pattern according to flow waveforms.

Breathing Pattern	DE	TDE	SQ	SIN	*p*
Respiratory rate, bpm	16 (15–17)	16 (15–17)	16 (15–17)	16 (15–17)	*0.397*
Tidal volume, mL	465 (428–500)	465 (428–500)	465 (428–500)	465 (428–500)	*0.735*
Minute ventilation, L/min	7.3 (6.7–8.3)	7.3 (6.7–8.3)	7.3 (6.7–8.3)	7.3 (6.7–8.3)	*0.155*
Total breathing time, s	3.7 (3.5–4.0)	3.7 (3.5–4.0)	3.7 (3.5–4.0)	3.7 (3.5–4.0)	*0.397*
Inspiratory time, s	1.1 (1.0–1.1)	1.1 (1.0–1.1)	1.1 (1.0–1.1)	1.1 (1.0–1.1)	*0.389*
Inspiratory Peak flow, mL/s	696 (622–761)	480 (428–539) *	411 (364–459) *^,#^	611 (533–494) *^,^°^,#^	***<0.001***
Mean Inspiratory flow, L/min	23.7 (20.8–26.1)	23.0 (19.4–24.7) *	21.8 (19.7–24.8) *	23.9 (21.3–26.4) °^,#^	***0.020***

DE: Decelerating Waveform; TDE: Trunk Decelerating Waveform; SQ: Square Waveform; SIN: Sinusoidal Waveform. *: vs. DE; °: vs. TDE; #: vs. SQ. *p* < 0.05. bpm: breath per minute.

**Table 3:** Respiratory mechanics and end-expiratory lung volume according to flow waveforms.

Respiratory Mechanics Variables	DE	TDE	SQ	SIN	*p*
Airway Peak pressure, cmH_2_O	28.5 (25.0–33.2)	27.8 (23.8–31)	28.5 (25–32.2) °	28.4 (25.8–33.8) °	***<0.001***
Airway Plateau pressure, cmH_2_O	20.3 (18.4–22.9)	20.2 (17.9–22.3)	20.0 (18.2–22.2) *	20.8 (18.2–22.2)	***0.008***
Driving pressure, cmH_2_O	8.9 (7.7–11.0)	8.9 (7.8–10.9)	8.7 (7.5–10.3)	8.7 (7.9–10.8)	*0.082*
Mean airway pressure, cmH_2_O	17 (15–18)	16 (14–17)	15 (14–17)	16 (14–16)	*0.276*
Respiratory system elastance, cmH_2_O/L	19.5 (15.8–26.1)	19.1 (15.3–25.4)	18.1 (15.5–23.3)	19.9 (15.8–25.0)	*0.195*
Lung elastance, cmH_2_O/L	13.1 (9.6–17.2)	12.6 (9.8–15.8)	11.9 (9.1–15.5)	12.4 (10.0–16.9)	*0.132*
Chest wall elastance, cmH_2_O/L	7.2 (5.4–8.6)	6.5 (4.7–8.4)	6.7 (6.0–8.3)	6.9 (5.0–8.7)	*0.412*
Mechanical power, J/min	17.5 (12.8–19.7)	17.0 (13.4–18.3)	16.7 (14.2–19.7) °	18.2 (14.0–20.8) °	***0.002***
End-expiratory lung volume, mL	1269 (787–1621)	1172 (829–1658)	1172 (729–1608)	1249 (829–1671)	*0.998*

DE: Decelerating Waveform; TDE: Trunk Decelerating Waveform; SQ: Square Waveform; SIN: Sinusoidal Waveform. *: vs. DE; °: vs. TDE; #: vs. SQ. *p* < 0.05.

**Table 4:** Gas exchange and right-to-left shunt according to flow waveforms.

Gas Exchange Variables	DE	TDE	SQ	SIN	*p*
PaO_2_, mmHg	73 (68–88)	71 (67–80)	70 (68–79) *	73 (68–83)	***0.039***
PaO_2_/FiO_2_, mmHg	181 (156–242)	176 (156–218)	176 (152–218) *	172 (152–211)	***0.030***
Right-to-left shunt, %	32 ± 8	36 ± 12	36 ± 10	36 ± 9 *	***0.004***
PaCO_2_, mmHg	47 (42–56)	47 (43–58)	50 (43–58) *	47 (43–61) *	***0.003***

DE: Decelerating Waveform; TDE: Trunk Decelerating Waveform; SQ: Square Waveform; SIN: Sinusoidal Waveform. *: vs. DE; °: vs. TDE; #: vs. SQ. *p* < 0.05. PaCO_2_: carbon dioxide arterial partial pressure; PaO_2_: oxygen arterial partial pressure; PaO_2_/FiO_2_: oxygen arterial partial pressure on inspired fraction of oxygen ratio.

## Data Availability

The data presented in this study are available on request from the corresponding author.
